# Supplementary material for: Tip60- and sirtuin 2-regulated MARCKS acetylation and phosphorylation are required for diabetic embryopathy
Source: Nat Commun. 2019 Jan 17;10:282. doi: 10.1038/s41467-018-08268-6 (PMC6336777; doi:10.1038/s41467-018-08268-6)

Supplementary information

# Tip60 and Sirtuin 2-regulated MARCKS acetylation and phosphorylation are required for diabetic embryopathy

Penghua Yang[1], Cheng Xu[1], Xi Chen[1], Min Zhan[2], Deborah J. Stumpo[3], Perry J. Blackshear[3, 4], E. Albert Reece[1, 5], Peixin Yang[1, 5]

[1]Department of Obstetrics, Gynecology & Reproductive Sciences, [2]Department of Epidemiology and Public Health, [5]Department of Biochemistry & Molecular Biology, University of Maryland School of Medicine Baltimore, Maryland, USA.
[3]Signal Transduction Laboratory, National Institute of Environmental Health Sciences, Research Triangle Park, NC 27709; and the [4]Departments of Medicine and Biochemistry, Duke University Medical Center, Durham, NC 27710

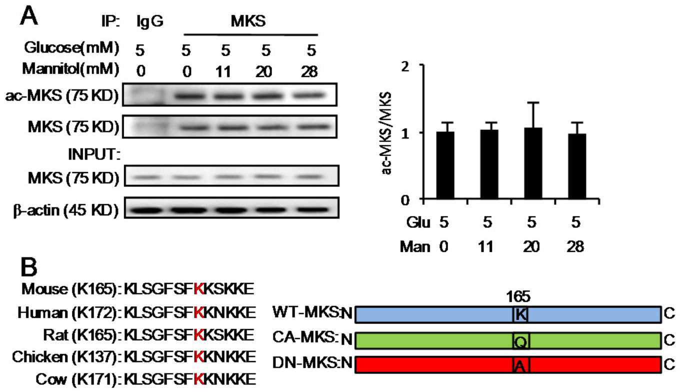


**Supplementary Figure 1. Mannitol does not induce MARCKS acetylation and MARCKS acetylation site mutation strategy.**

(**A)** Abundance of acetylated MARCKS (ac-MKS) in cultured cells with mannitol, a glucose osmotic control. All samples were pulled down by the rabbit anti-MARCKS antibody. The incubation time of high glucose was 48 h. IP: Immunoprecipitation; INPUT: 30 μg protein each group was loaded per lane; MKS: MARCKS. All experiments were repeated three times (N = 3). Quantification data were shown in the bar graph. One way ANOVA followed by the *Tukey* test was used to analyze the data. **(B)** Acetylation site at lysine 165 of the mouse *Marcks* gene was mutated into glutamine (Q) to mimic acetylation or alanine (A) for blocking acetylation.

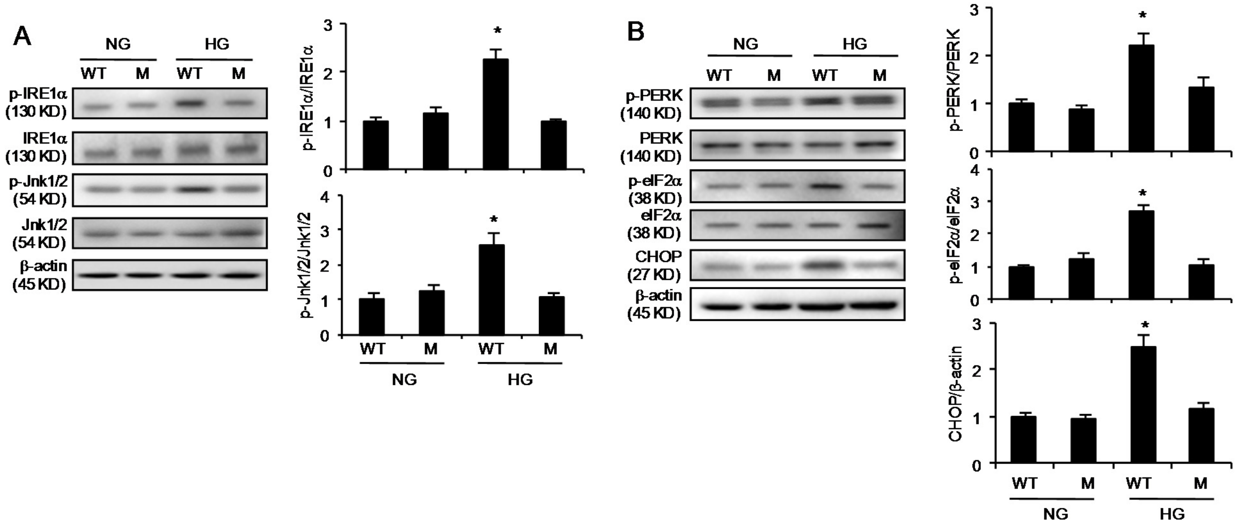


**Supplementary Figure 2. MARCKS-PD suppresses high glucose-induced ER stress.**

**(A)** Protein abundance of p-IRE1α and p-JNK1/2 in cultured cells. **(B)** Abundance of p-PERK, p-eIF2α and CHOP in cultured cells. Quantification data was shown in the bar graphs. M: MARCKS-PD, NG: normal glucose (5 mM), HG: high glucose (25 mM). All experiments were repeated three times (N = 3). One way ANOVA followed by the *Tukey* test was used to analyze the data. * indicates significant difference ($P < 0.05$) compared to other groups.

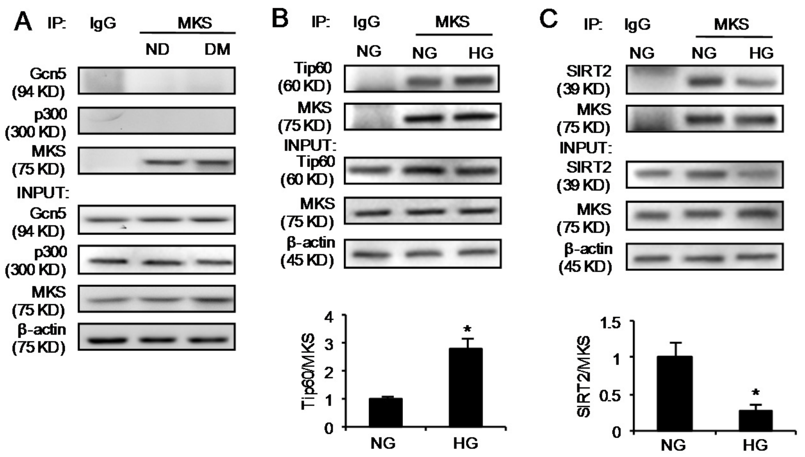


**Supplementary Figure 3. Tip60 binds to MARCKS and high glucose reduces the interaction between SIRT2 and MARCKS.**

**(A)** Co-immunoprecipitation of MARCKS with Gcn5 and p300 in embryos. Three litters from three different dams (N = 3) were performed in each group. (**B**) Abundance of Tip60 and MARCKS (MKS) in cultured cells from immunoprecipitation (IP). **(C)** Abundance of SIRT2 and MARCKS (MKS) in cultured cells from IP. MKS: MARCKS, ND: nondiabetic, DM: diabetes mellitus. NG: normal glucose (5 mM), HG: high glucose (25 mM). All experiments were repeated three times (N = 3). Data analysis were performed by the *t* test. * indicates significant difference ($P < 0.05$) compared to the other group.

Supplementary Table 1. Overexpression of MARCKS-PD ameliorates maternal diabetes-induced NTDs

| | Experimental group | Glucose level (mg/dl) | Genotype | Total embryos | NTD embryos | NTD rate (%) |
|---|---|---|---|---|---|---|
| ND | MKS-PD ♂ ×WT ♀ (6 litters) | 130.8±22.4 | WT | 19 | 0 | 0 |
| | | | MKS | 21 | 0 | 0 |
| DM | MKS-PD ♂ ×WT ♀ (13 litters) | 379.9±73.2 | WT | 43 | 12 | 27.9* |
| | | | MKS | 39 | 3 | 7.7 |

NTDs: neural tube defects. ND: nondiabetic; DM: diabetic; MKS: MARCKS; WT: wild-type; ♂: male; ♀: female; * indicates significant difference when compared to other groups using *Chi*-square tests.

Supplementary Table 2. SIRT2 deficient embryos in the condition of pathogenic maternal diet treatment develop normally

| | Groups | Genotypes | Number of NTD embryos | Total embryos |
|---|---|---|---|---|
| Control | $S2^{+/-}$ x $S2^{+/-}$ (n = 4 litters) | WT | 0 | 7 |
| | | $S2^{+/-}$ | 0 | 17 |
| | | $S2^{-/-}$ | 0 | 8 |
| Purina5015 | $S2^{+/-}$ x $S2^{+/-}$ (n = 6 litters) | WT | 0 | 11 |
| | | $S2^{+/-}$ | 0 | 20 |
| | | $S2^{-/-}$ | 0 | 9 |

NTDs: neural tube defects. S2: SIRT2; +/+: wildtype; +/-: heterozygous; -/-: homozygous; Control: Purina5001 diet. Data analysis were performed using the *Chi*-square test.

Supplementary Table 3. The combination of SIRT2 deficiency and PKC activation induces NTDs in cultured embryos

| | Groups | Genotypes | Number of NTD embryos | Total embryos |
|---|---|---|---|---|
| Control | S2$^{+/-}$ x S2$^{+/-}$ | WT | 0 | 7 |
| | | S2$^{+/-}$ | 0 | 16 |
| | | S2$^{-/-}$ | 0 | 6 |
| ROPA | S2$^{+/-}$ x S2$^{+/-}$ | WT | 0 | 6 |
| | | S2$^{+/-}$ | 2(16.7%) | 12 |
| | | S2$^{-/-}$ | 5(55.6%)* | 9 |

NTDs: neural tube defects. S2: SIRT2; +/+: wildtype; +/-: heterozygous; -/-: homozygous; For each litter (total 9 litters from nondiabetic dams), embryos were randomly assigned to control (vehicle: water) and 1□M ROPA treatment respectively. * indicates significant difference when compared to the WT/ROPA treated group, and the Control groups using Fisher Exact tests.

Supplementary Table 4. SIRT2 overexpression in the neuroepithelium reduces maternal diabetes-induced NTDs

| | Experimental group | Glucose levels (mg/dl) | Genotype | Total embryos | NTD embryos | NTD rate (%) |
|---|---|---|---|---|---|---|
| ND | SIRT2♂×WT♀ （7 litters） | 155.1±12.7 | WT | 25 | 0 | 0 |
| | | | SIRT2 | 26 | 0 | 0 |
| DM | SIRT2♂×WT♀ （16 litters） | 394.7±82.8 | WT | 54 | 12 | 22.2* |
| | | | SIRT2 | 55 | 3 | 5.5 |

NTDs: neural tube defects; ND: nondiabetic; DM: diabetic; WT: wild-type; ♂: male; ♀: female; * indicates significant difference when compared to other groups using *Chi*-square tests.

Supplementary Table 5. Antibodies for immunoblotting

| Antibodies name | Antibody sources | Catalogue number | Dilution |
|---|---|---|---|
| MARCKS | Cell signaling technology | 5607 | 1:1000 |
| p-MARCKS | Cell signaling technology | 2741 | 1:1000 |
| Acetylation | Cell signaling technology | 9814 | 1:1000 |
| Tom20 | Santa Cruz | SC-11415 | 1:1000 |
| CNX | Sigma | SAB4503258 | 1:1000 |
| Puma | Cell signaling technology | 4976 | 1:1000 |
| Bim | Cell signaling technology | 2819 | 1:1000 |
| Bik | Cell signaling technology | 4592 | 1:1000 |
| Bak | Cell signaling technology | 12105 | 1:1000 |
| PHB | Cell signaling technology | 2426 | 1:1000 |
| p-IRE1α | Invitrogen | PA1-16927 | 1:1000 |
| IRE1α | Cell signaling technology | 3294 | 1:1000 |
| p-JNK1/2 | Cell signaling technology | 9251 | 1:1000 |
| JNK1/2 | Cell signaling technology | 9252 | 1:1000 |
| p-PERK | Cell signaling technology | 3179 | 1:1000 |
| PERK | Cell signaling technology | 3192 | 1:1000 |
| p-eIF2α | Cell signaling technology | 3597 | 1:1000 |
| eIF2α | Cell signaling technology | 2103 | 1:1000 |
| CHOP | Cell signaling technology | 5554 | 1:1000 |
| Cas3 | Millipore | 2722838 | 1:1000 |
| Cas8 | Millipore | AB1879 | 1:1000 |
| Sox1 | Abcam | AB87775 | 1:1000 |
| Tuj1 | Santa cruz | SC-80016 | 1:1000 |
| Tip60 | Thermo Scientific | PA5-29457 | 1:1000 |
| SIRT2 | Cell signaling technology | 12672 | 1:1000 |
| H4K16ac | Millipore | DAM1805250 | 1:1000 |
| H4 | Cell signaling technology | 2935BC | 1:1000 |

Supplementary Table 6. PCR Primers for genotyping

| Primers | Sequence (5'-3') |
|---|---|
| XBP1 splicing forward | GAACCAGGAGTTAAGAACACG |
| XBP1 splicing reverse | AGGCAACAGTGTCAGAGTCC |
| β-actin forward | GTGACGTTGACATCCGTAAAGA |
| β-actin reverse | GCCGGACTCATCGTACTCC |
| Nestin-Sirt2 genotyping forward | GGGCCACTCCCTTCTCTAGT |
| Nestin-Sirt2 genotyping reverse | TGTAGCGTGTCACTCCTTCG |
| Sirt2 knockout genotyping forward#1 | GACTGGAAGTGATCAAAGCTC |
| Sirt2 knockout genotyping forward#2 | CAGGGTCTCACGAGTCTCATG |
| Sirt2 knockout genotyping reverse | TCAAATCTGGCCAGAACTTATG |
| Marcks transgene genotyping forward | CTCGACTTCTTCGCCCAA |
| Marcks transgene genotyping reverse#1 | TAGTCTGGGACGTCGTATGG |
| Marcks transgene genotyping reverse#2 | TGGGACGTCGTATGGGTA |

**Supplementary Figure 4. Uncropped immunoblotting images.**

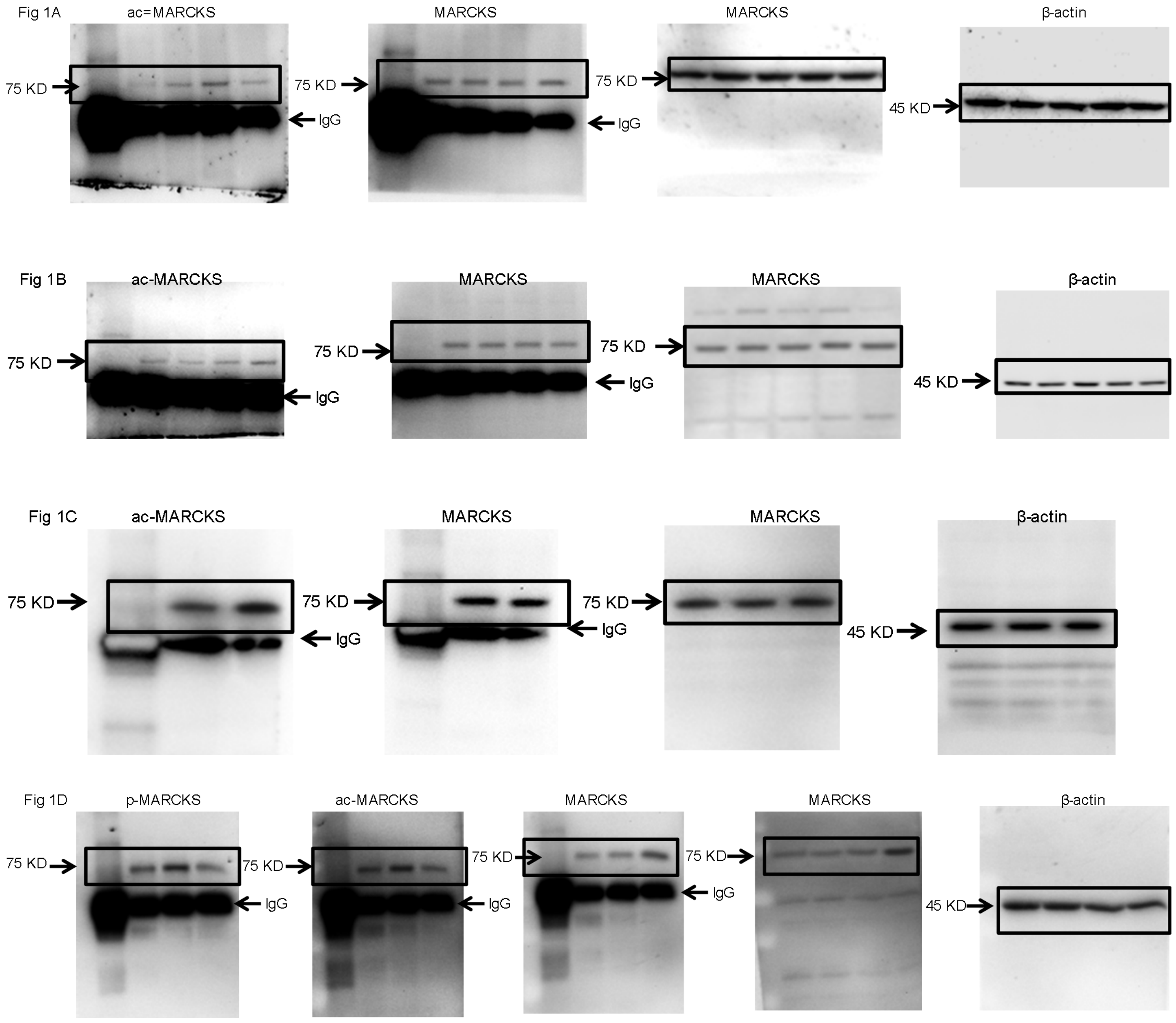

Fig 1E

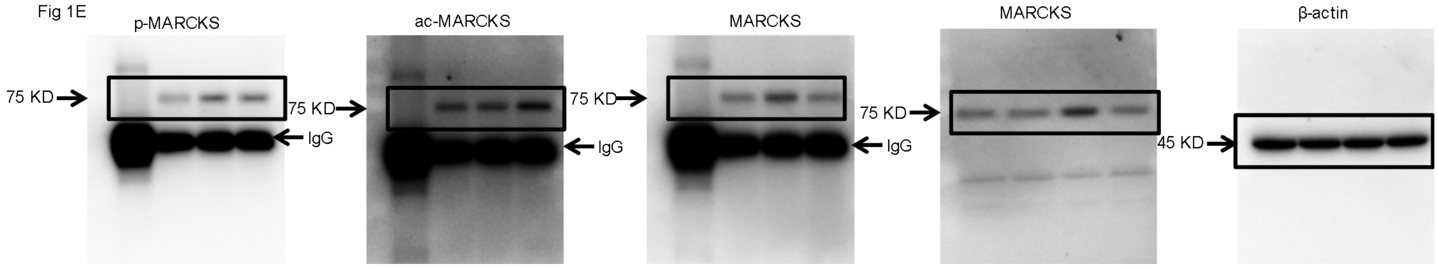


Fig 1F

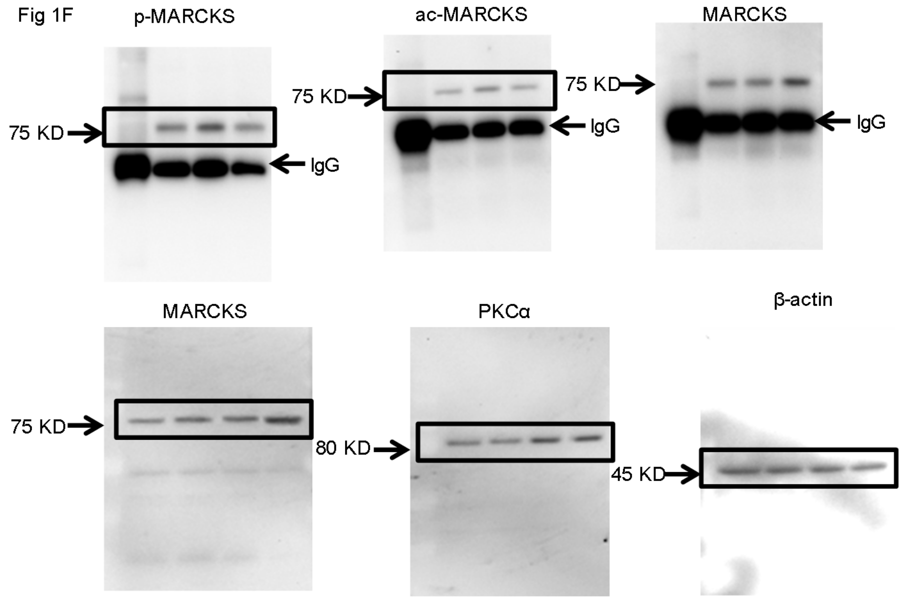


Fig 2A

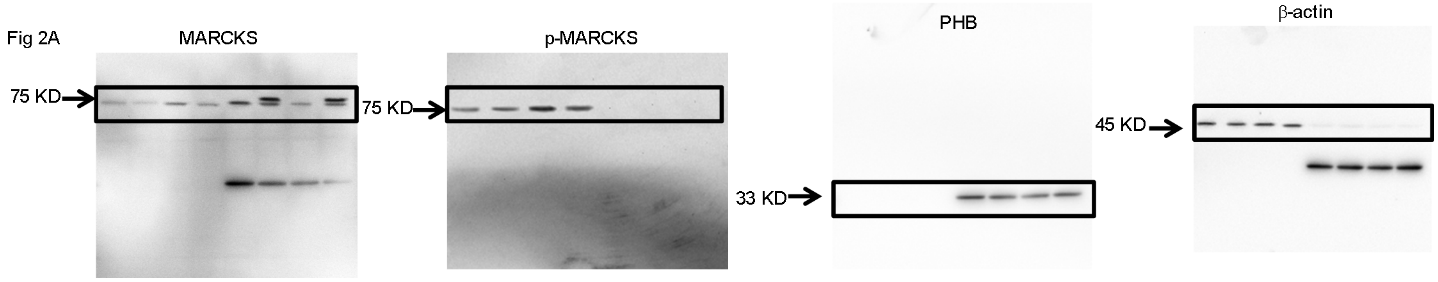


Fig 2B

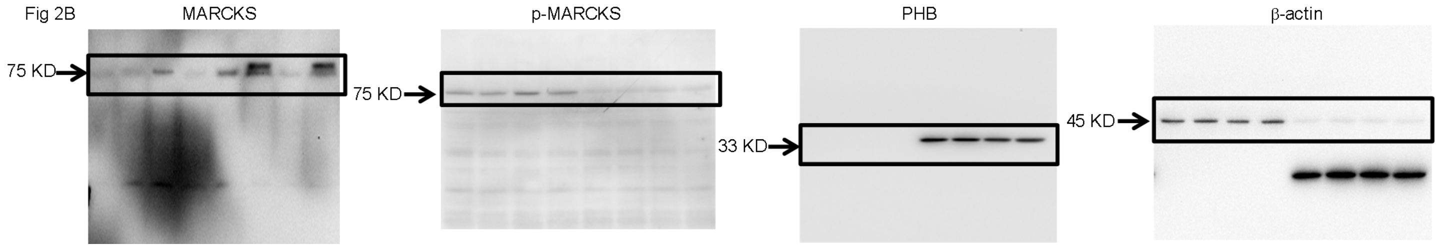

Fig 3B

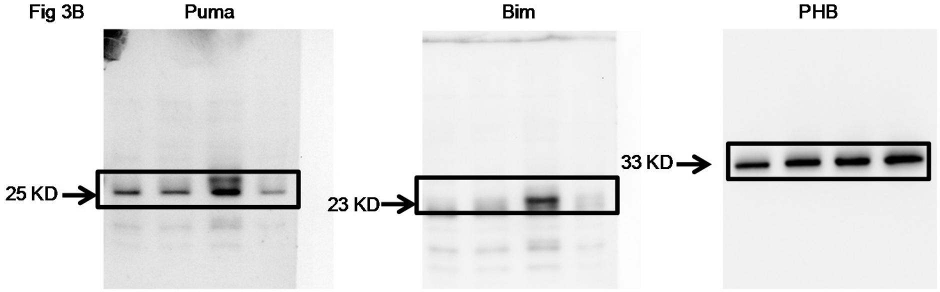


Fig 3C

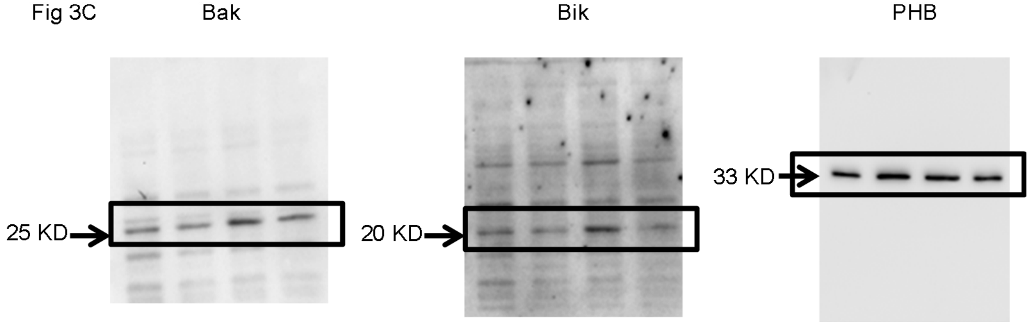


Fig 3D

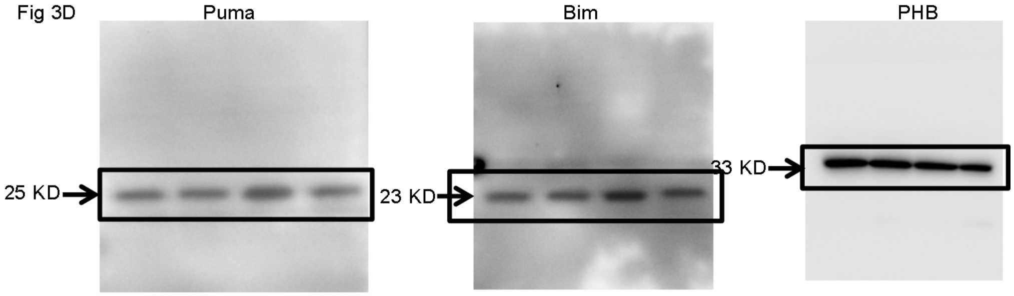


Fig 3E

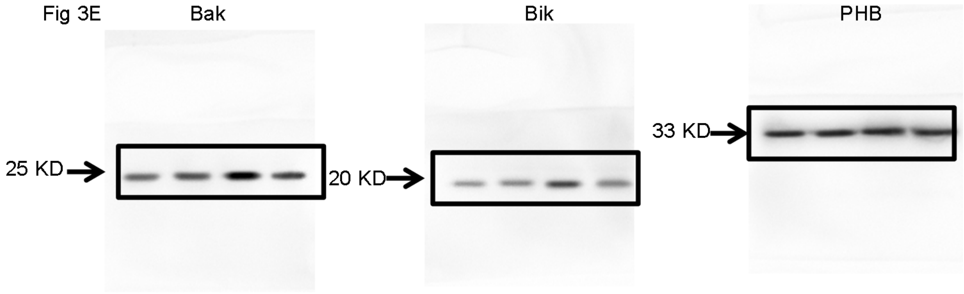


Fig 4E

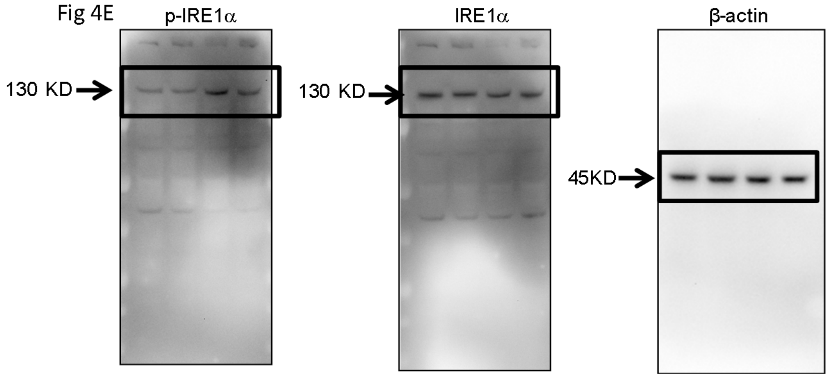

Fig 4F

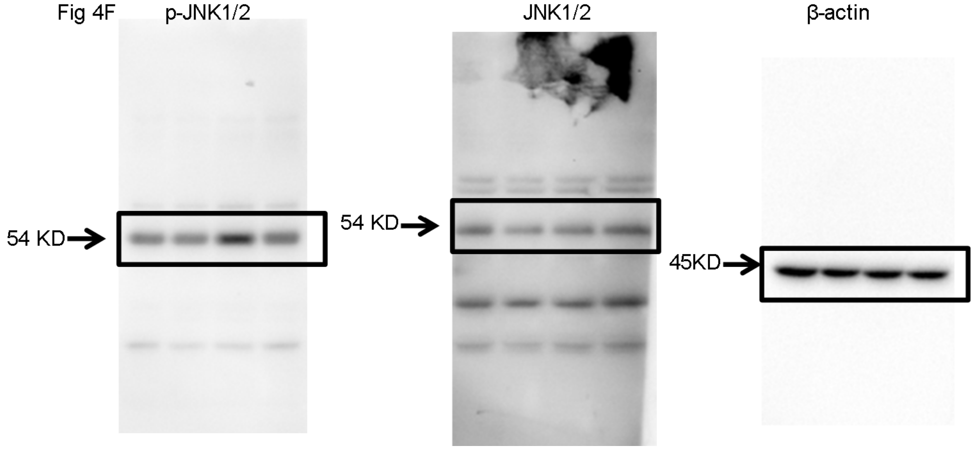


Fig 4H

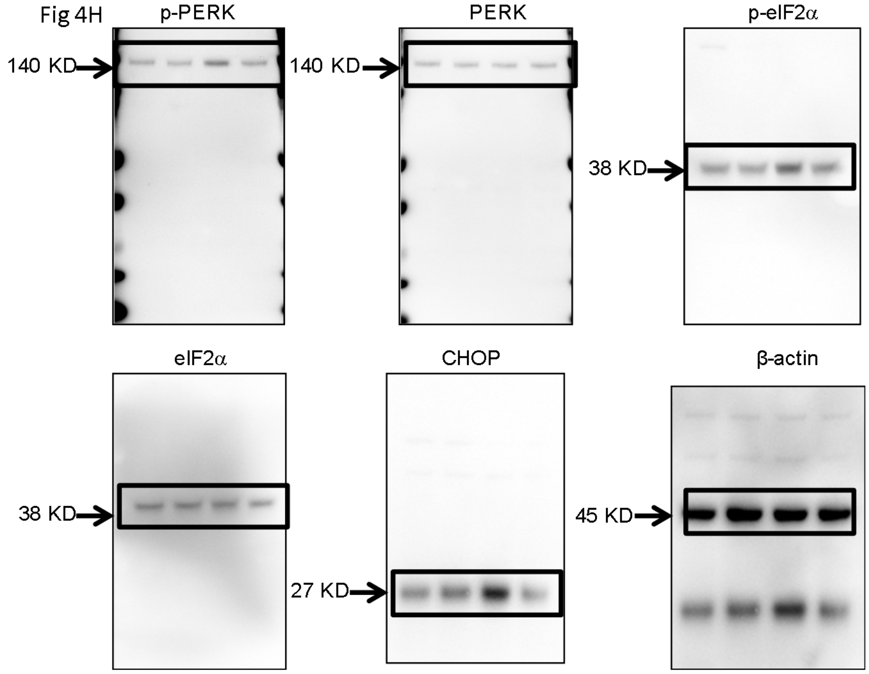


Fig 5B

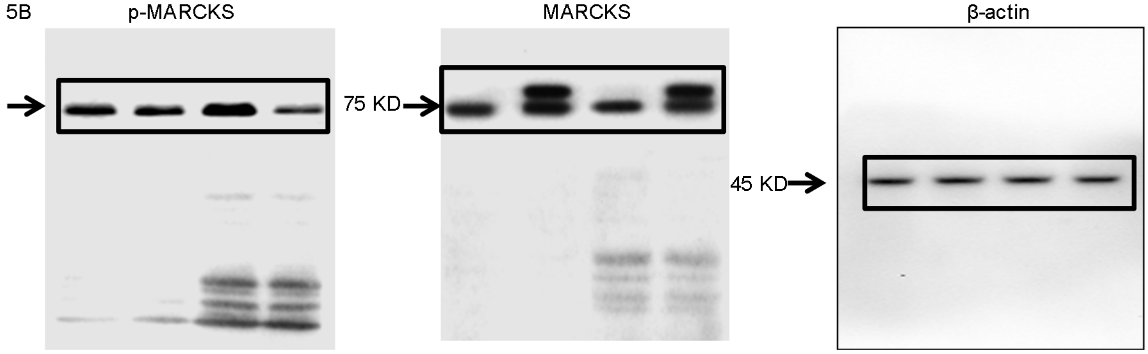

Fig 5E

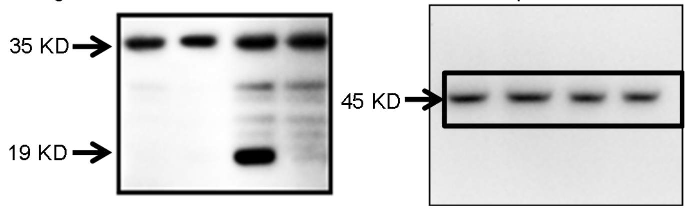


Fig 5F

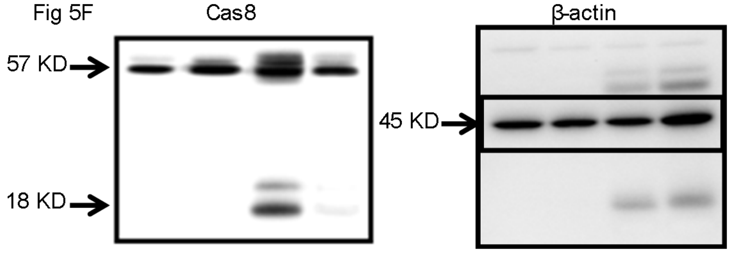


Fig 6A

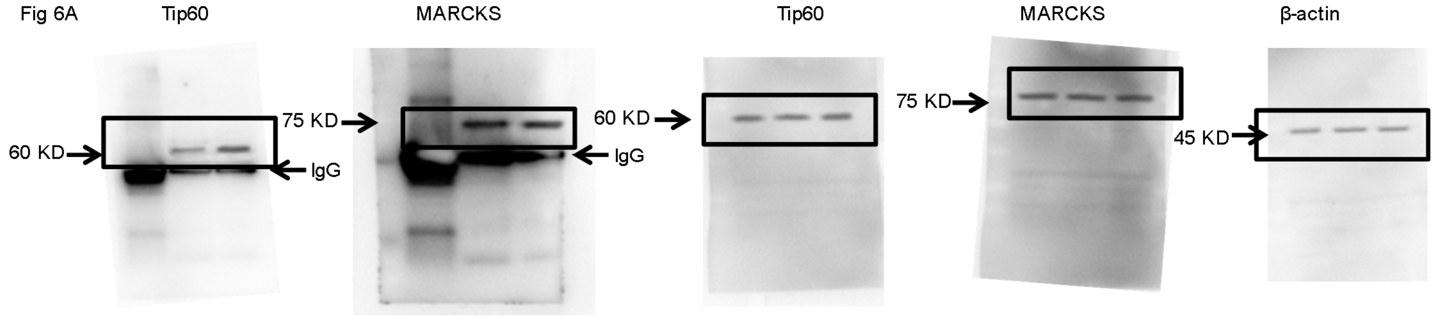


Fig 6B

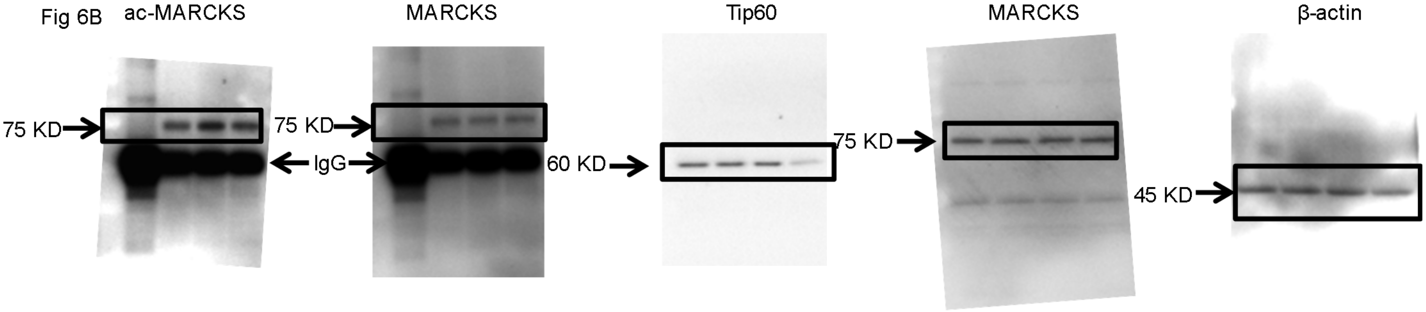


Fig 6C

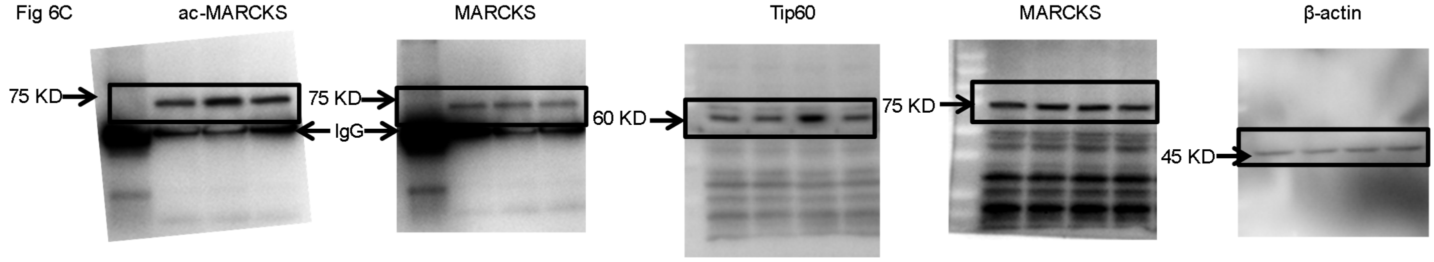

Fig 6D

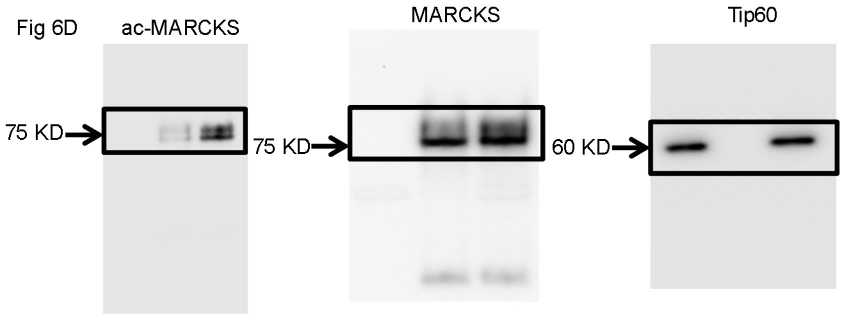


Fig 6F

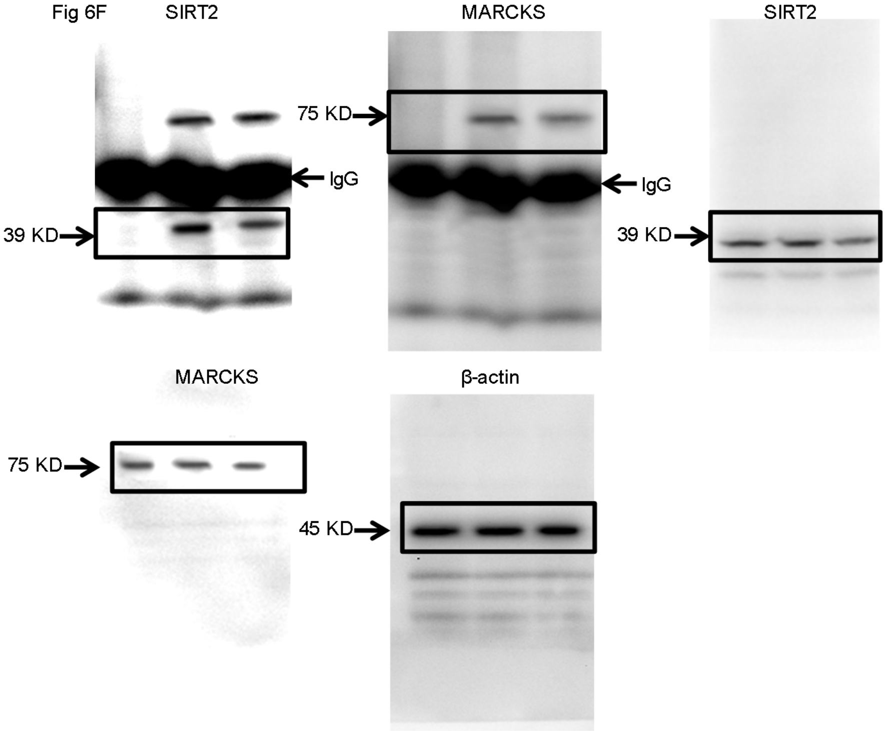


Fig 6G

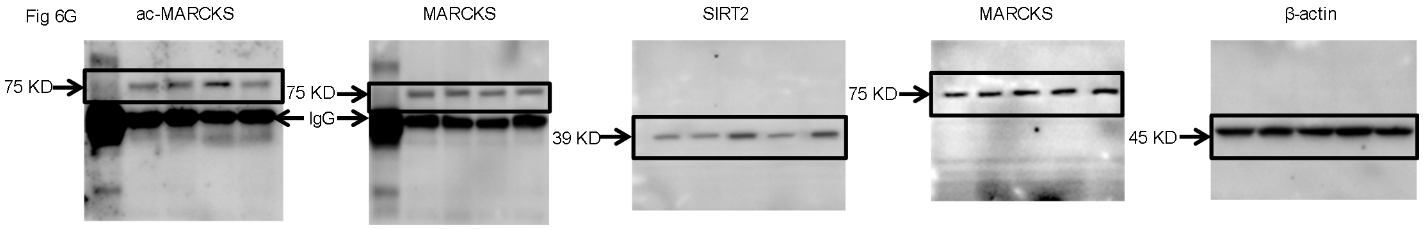


Fig 6H

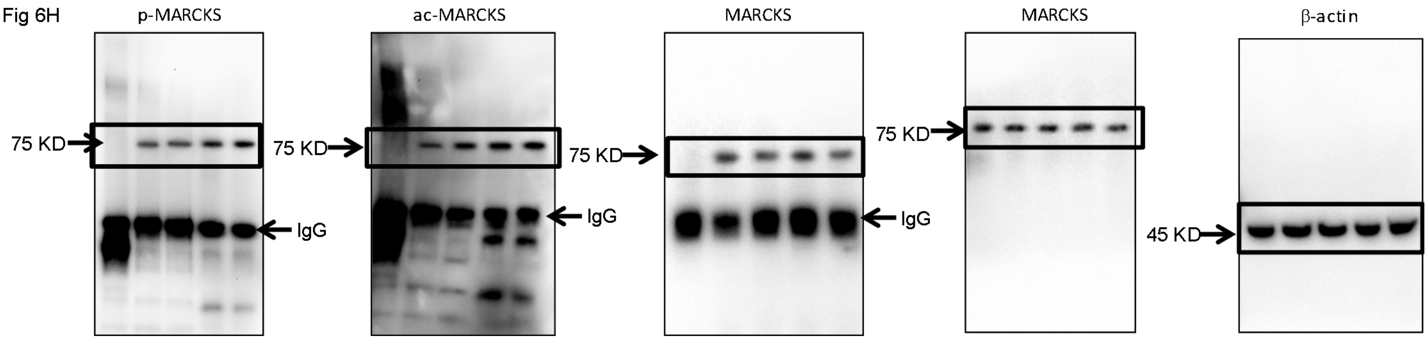

Fig 6I

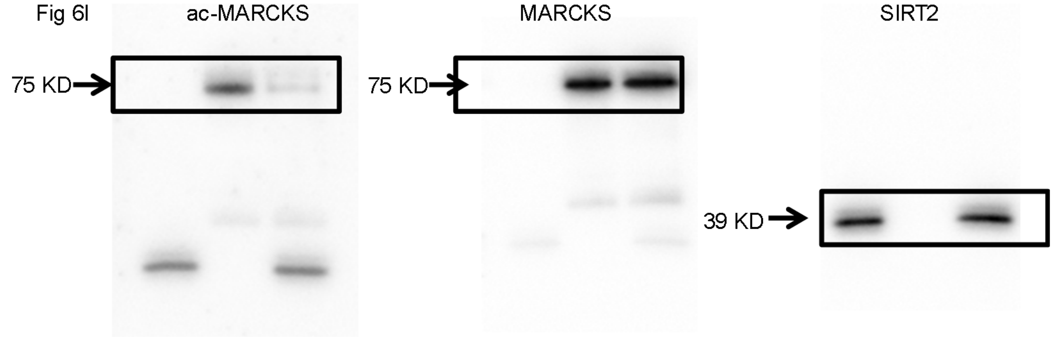


Fig 6J

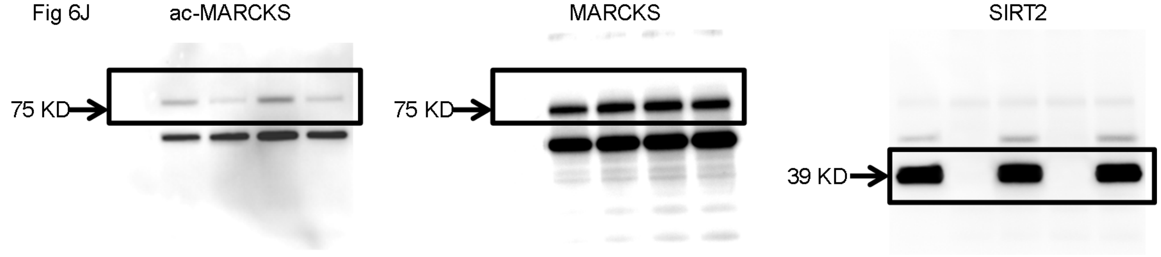


Fig 6K

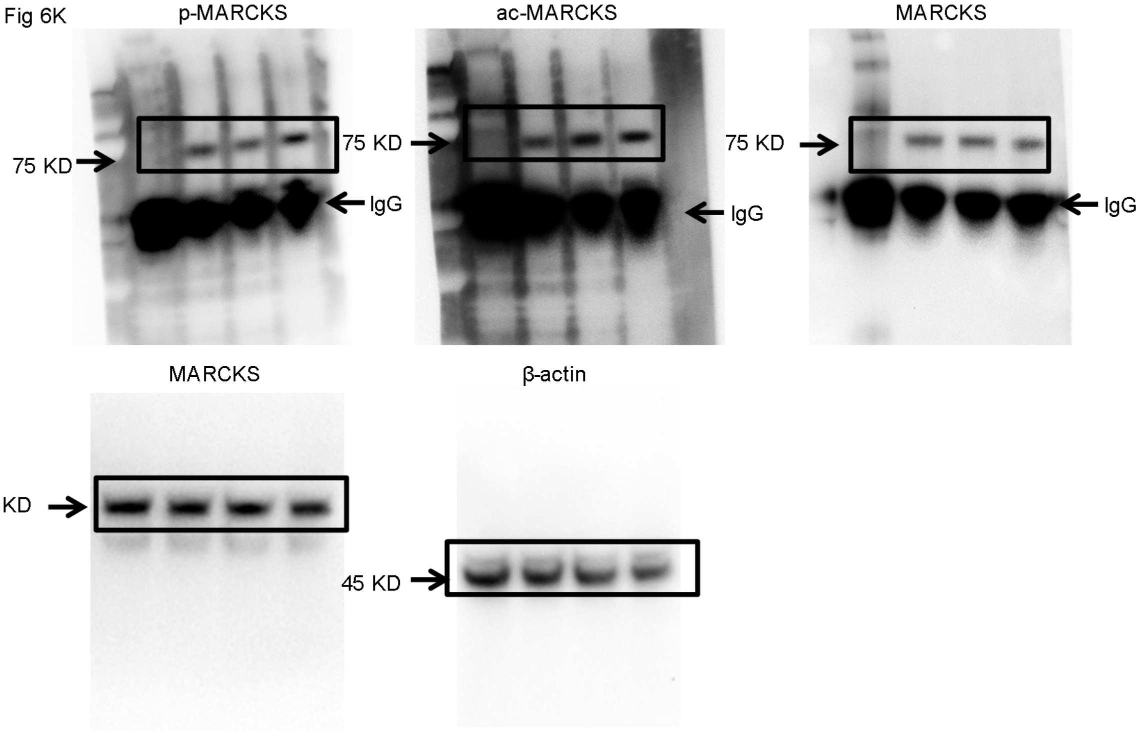


Fig 6M

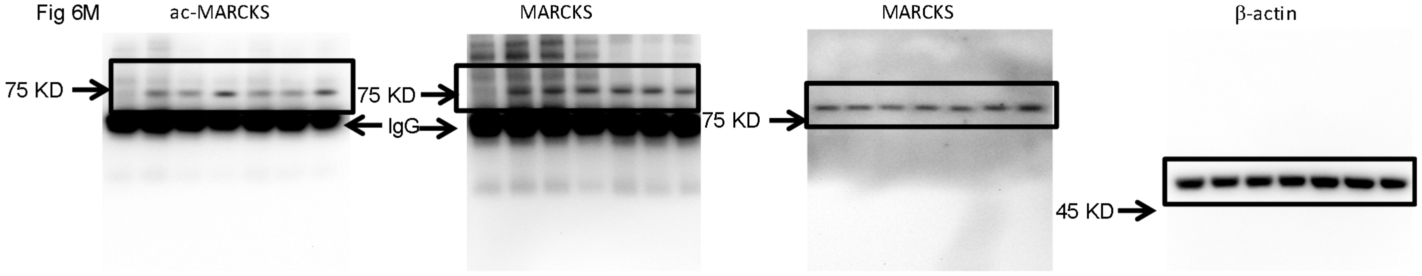

Fig 6N

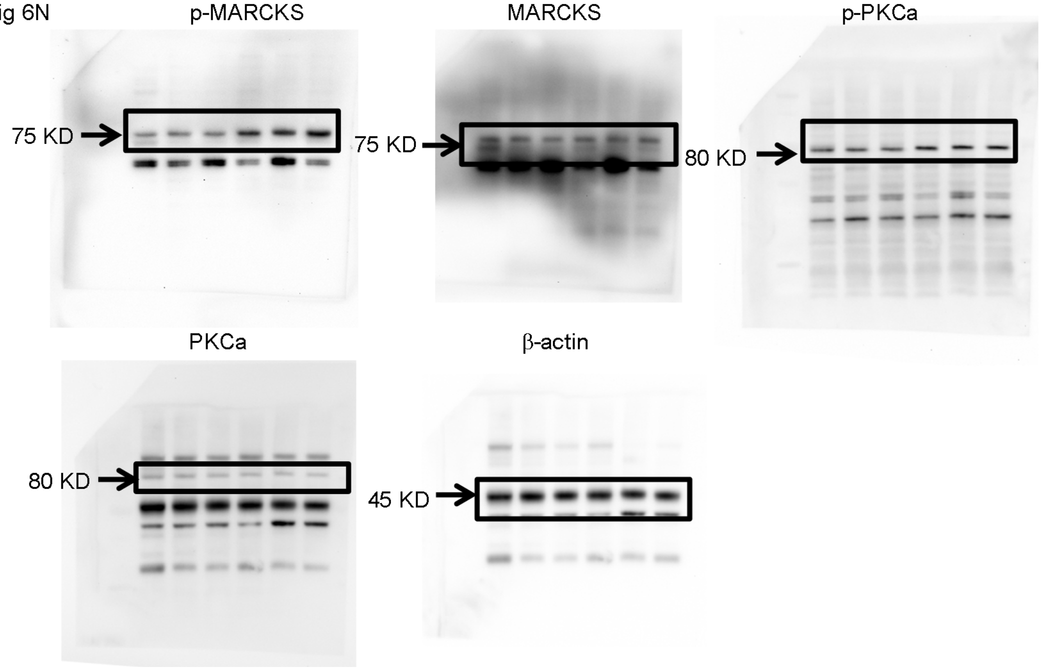


Fig 7C

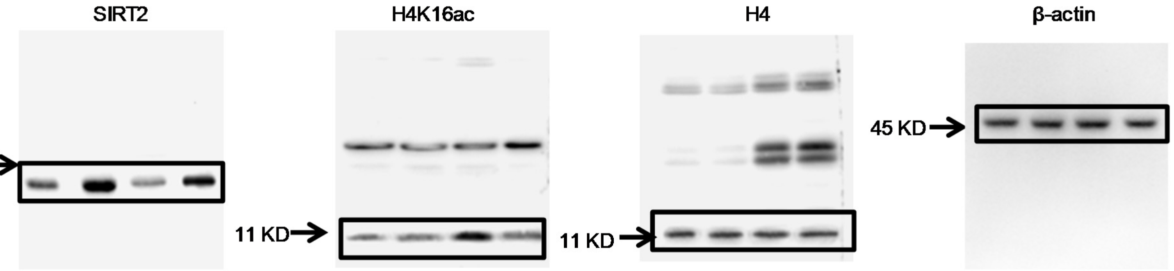


Fig 7G

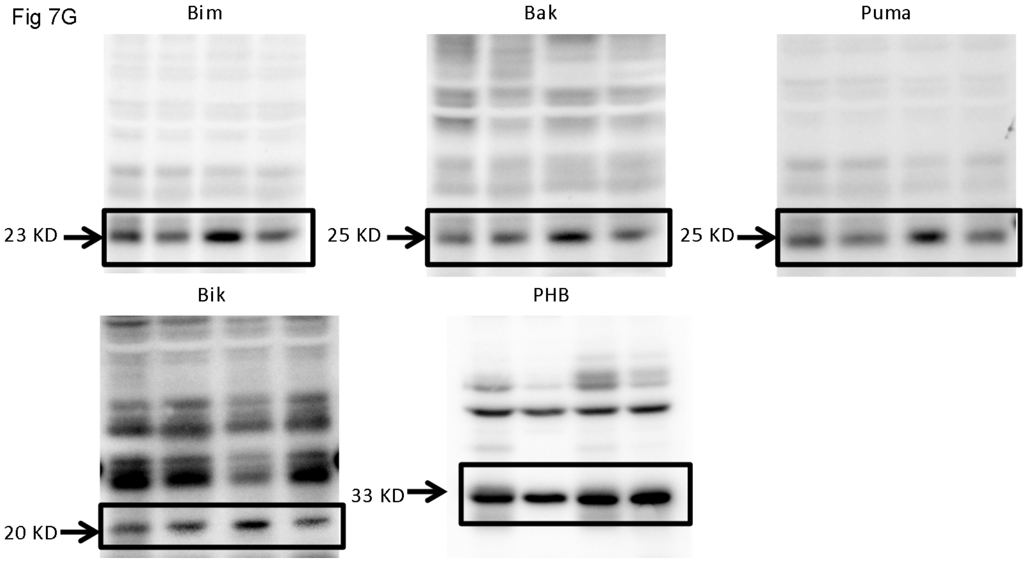

Fig 7H

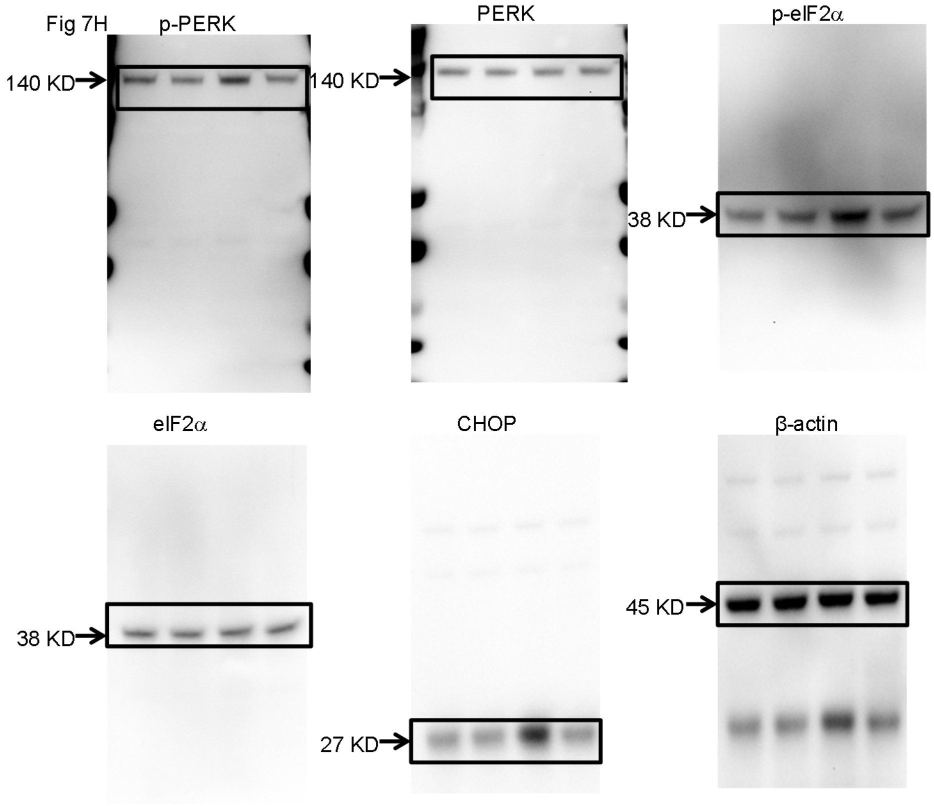


Fig 7I

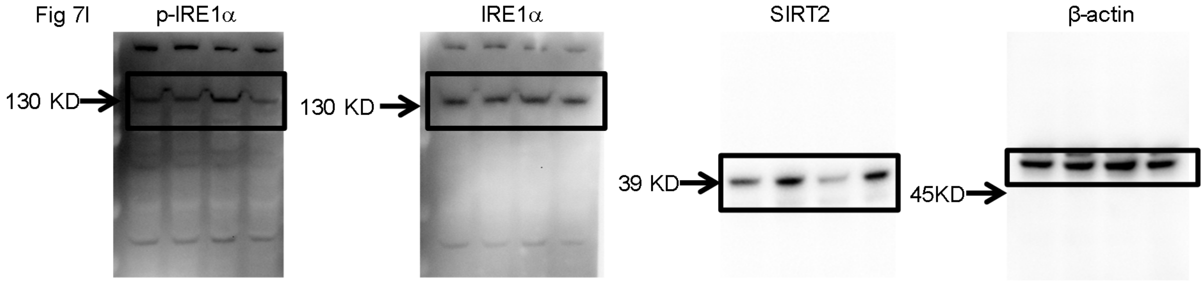


Fig S1A

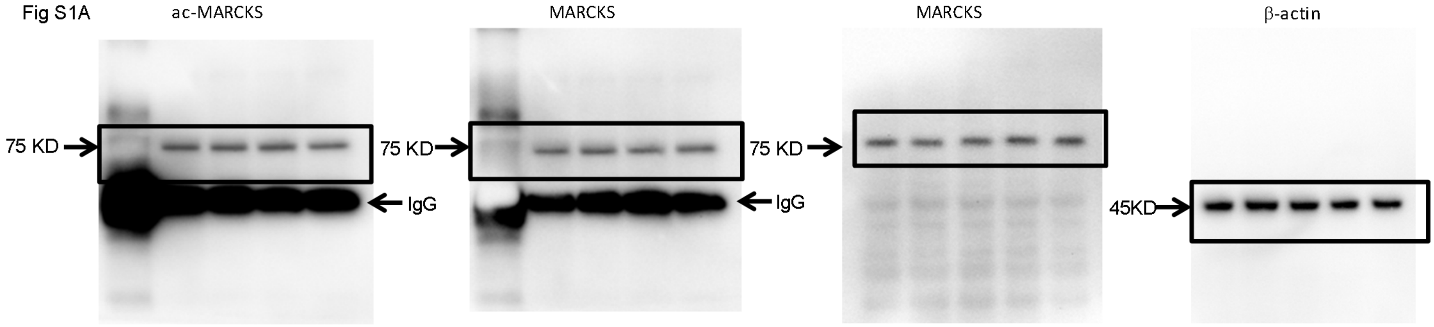

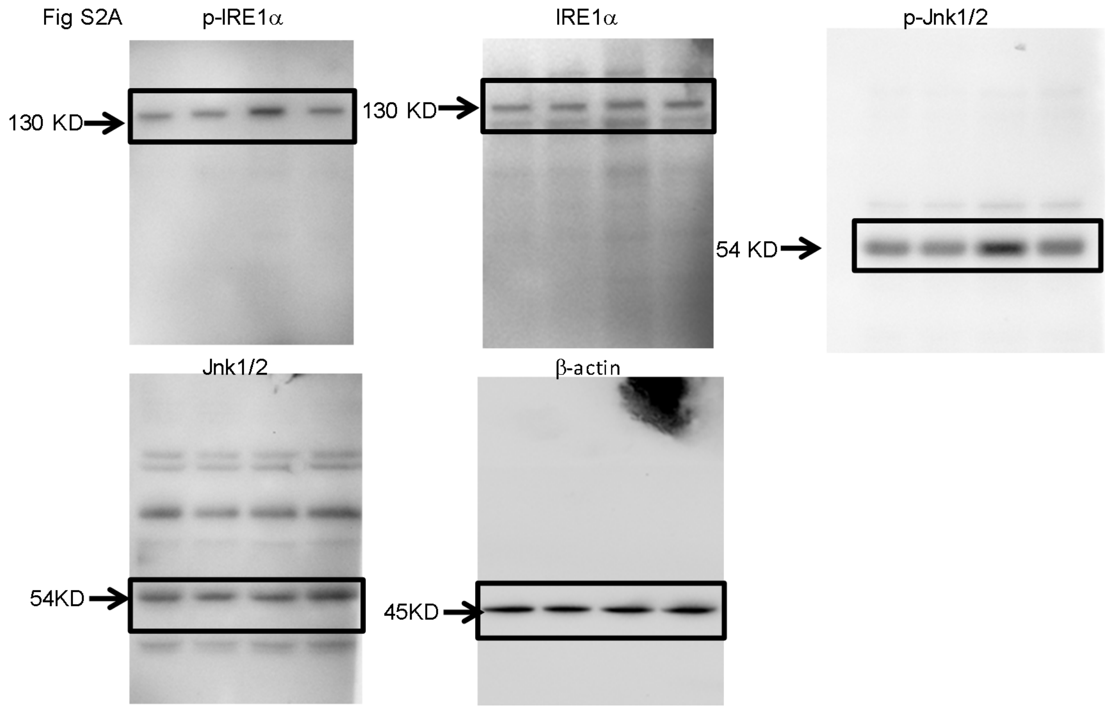
Fig S2A
p-IRE1α
IRE1α
p-Jnk1/2
130 KD
130 KD
54 KD
Jnk1/2
β-actin
54KD
45KD

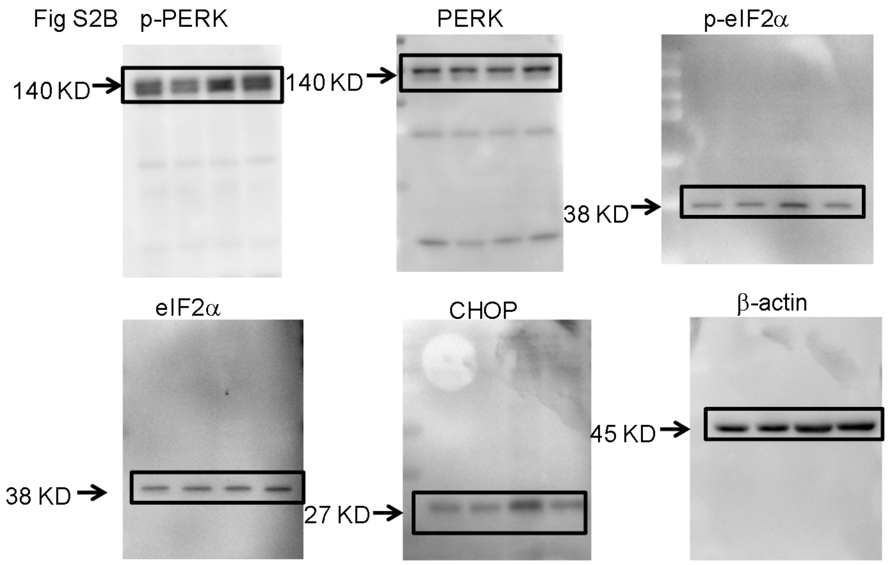
Fig S2B
p-PERK
PERK
p-eIF2α
140 KD
140 KD
38 KD
eIF2α
CHOP
β-actin
45 KD
38 KD
27 KD

Fig S3A

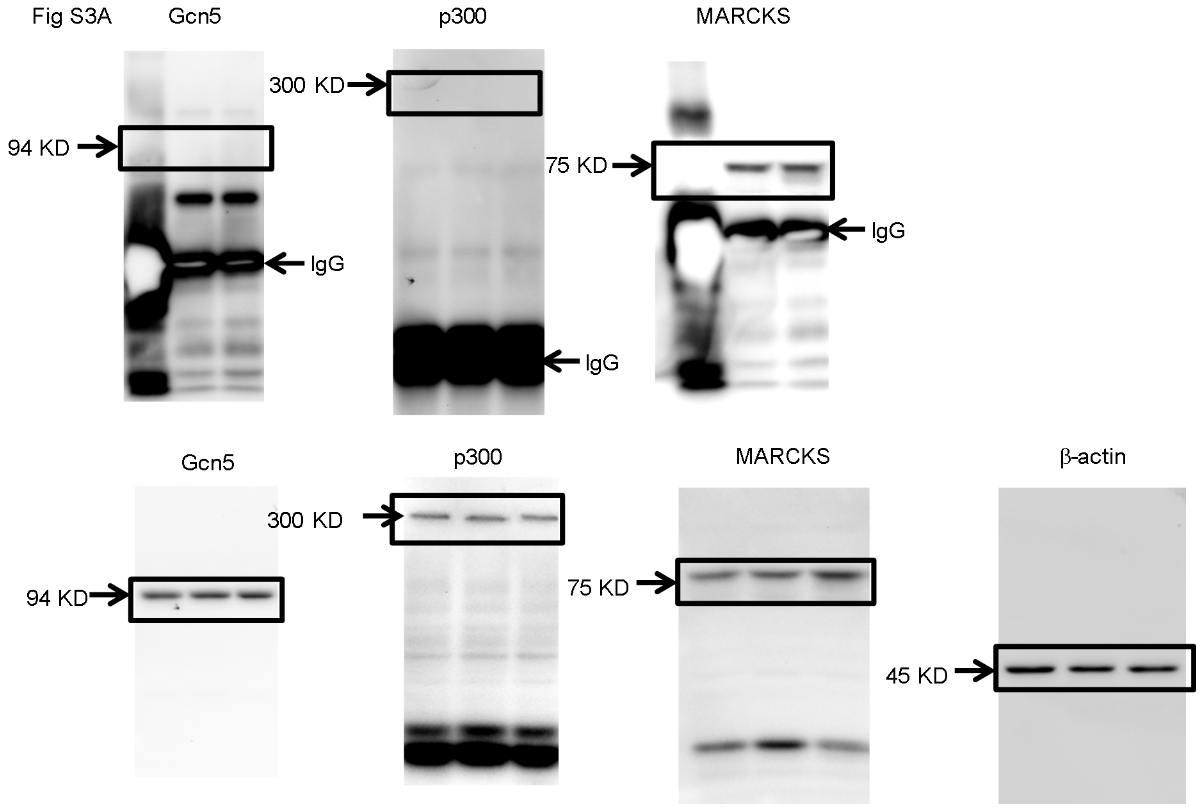


Fig S3B

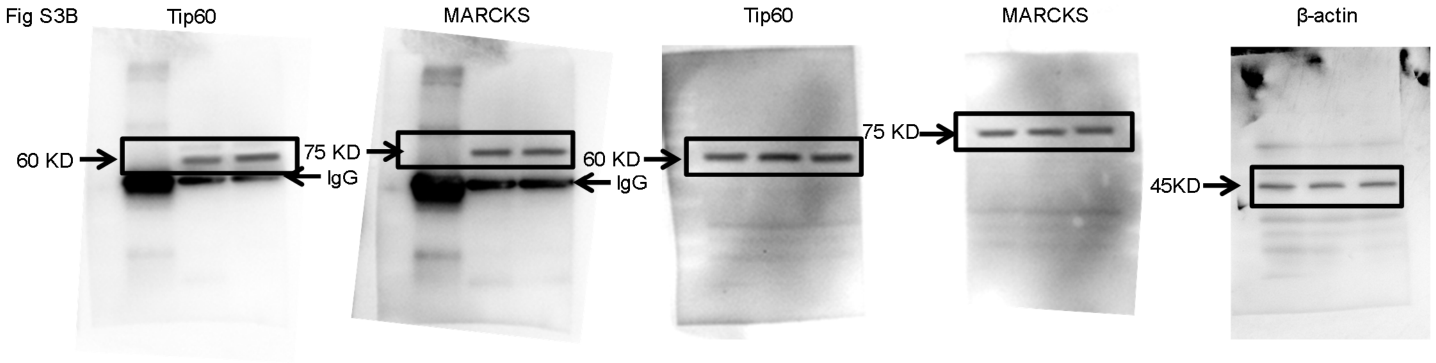


Fig S3C

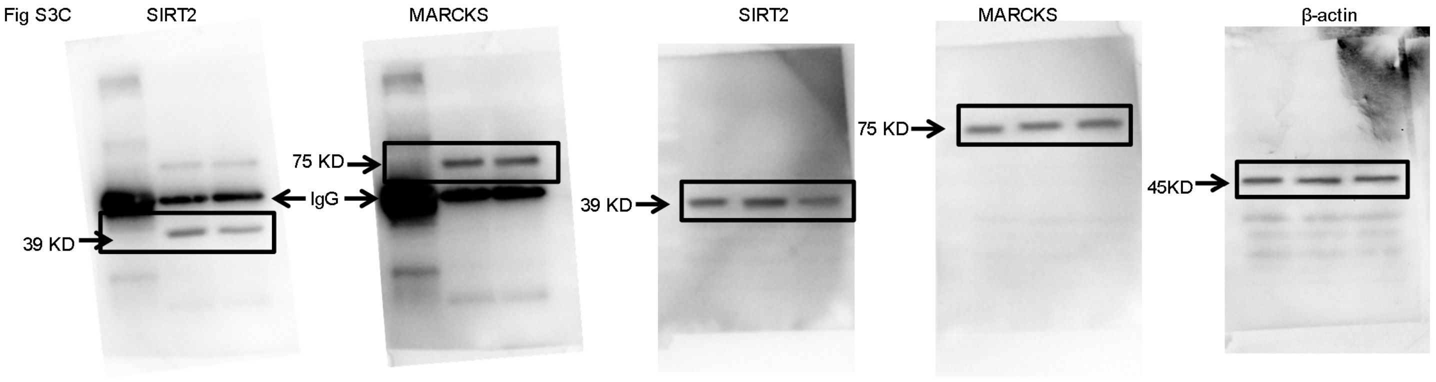

Supplement: Supplementary file 1 — Supplementary Information [file 41467_2018_8268_MOESM1_ESM.pdf]
